# Data on the impact of socioeconomic status on academic achievement among students in Malaysian public universities

**DOI:** 10.1016/j.dib.2020.106018

**Published:** 2020-07-14

**Authors:** Nor Fatimah Che Sulaiman, Noor Haslina Mohamad Akhir, Nor Ermawati Hussain, Rahaya Md Jamin, Nur Hafizah Ramli

**Affiliations:** a Faculty of Business, Economics and Social Development, Universiti Malaysia Terengganu, Terengganu, Malaysia; b Institute of Tropical Biodiversity and Sustainable Development, Universiti Malaysia Terengganu, Terengganu, Malaysia

**Keywords:** Performance, Academic qualification, Employment sector, Income level, Universiti Malaysia Terengganu

## Abstract

This data article presents the impact of parents' socioeconomic status on undergraduate students’ academic achievements at a Malaysian higher education institution. The eastern parts of Peninsular Malaysia are populated by low-income citizens compared to the national average. The survey was conducted in Universiti Malaysia Terengganu. The targeted population is final year social science students. The total size of the target population is 965 students. Using Krejcie and Morgan's sampling method, a sample size of 333 students was surveyed. A descriptive research design was adopted in this study. Data were obtained from stratified random sampling comprising a total of 333 respondents in Universiti Malaysia Terengganu from 14 states across Malaysia. The data were collected through a semi-structured questionnaire. Data analysis was carried out using tables and figures. The findings revealed that most of the students stated that a parent's socioeconomic status does not influence their academic achievement.

**Specifications table**

**Table:** 

**Subject**	Economics
**Specific subject area**	Economic Development
**Type of data**	Table
	Figure
	Text
**How data were acquired**	Questionnaire Survey
**Data format**	Raw and analysed data
	Descriptive statistics
**Parameters for data collection**	Gender, age, race, marital status, state, academic qualification, socioeconomic status of parent and student academic achievement
**Description of data collection**	Data were gathered through questionnaires distributed to student in Universiti Malaysia Terengganu using stratified random sampling. Data were screened for missing values and outliers were checked before pursuing the data analysis. Normality and reliability tests were also conducted before the descriptive and presented as frequency and percentage.
**Data source location**	Data were collected in Universiti Malaysia Terengganu located in Terengganu with multicultural students from diverse socioeconomic backgrounds from all states in Malaysia; Johor, Kedah, Kelantan, Melaka, Negeri Sembilan, Pahang, Pulau Pinang, Perak, Perlis, Selangor, Terengganu, Sabah, Sarawak and Wilayah Persekutuan Kuala Lumpur.
**Data accessibility**	All data in this data article are contained in the supplementary data file.

**Value of the Data**

• The data provides important information about university students’ academic achievement and can be used to further understand the relationship between the parent's socioeconomic status and student's academic achievement. These findings could be useful for other countries.
• The dataset will help stimulate in-depth research on the academic achievement of students in Malaysian universities and can be implemented to students at the school level. It will provide valuable and meaningful information and tool for analysis at the university level, school level, policymakers and other stakeholders in terms of socioeconomic status and academic achievement.
• The dataset could be analysed further using more advanced analysis and develop further experiments applying bigger sample data of universities across Malaysia.
• The data are important for the policy makers, planners, and managers to make predictions of the needs for the higher education requirements several years ahead.

## 1 Data

This data can contribute to strengthening data readiness and filling data gaps to develop a comprehensive dataset for implementing the Sustainable Development Goal (SDG) by 2030. Malaysia is looking forward to achieving SDG Goal 4 regarding Quality Education, which aims to ensure inclusive and equitable quality education and promote lifelong learning opportunities for all. Target 4.3 ensures equal access for all women and men to affordable and quality technical, vocational, and tertiary education, including university by 2030 [1].

This data highlights the relationship of student achievement and its contingency on one's socioeconomic situation to support human capital growth towards better economic development. This paper introduces the impact of socioeconomic status (SES) on academic achievement. Academic achievement can measure a student's level of proficiency in a given field. Academic achievement is seen as one of the key indicators for employers to recruit. Therefore, the issue of academic achievement amongst university students is critical as human capital can shape and develop the country's economy. Hassan and Rasiah (2011) [2] and Abdullah [3] found that parents who spend on education produce students with better educational outcomes. Fundamentally, human capital is linked to the level of knowledge, technical skills, creativity, and experience [4]. The level of knowledge is often measured from an individual's level of education. Veas, Gilar and Miñano (2016) [5] found that academic achievement is linked to intellectual ability. Shaarani et al. (2015) [6] found that parental support factors also play an important role in improving student academic achievement.

The survey was conducted through semi-structured questionnaires to Universiti Malaysia Terengganu's student from all states in Malaysia. The states were then divided into the Eastern Region, Western Region, Northern Region, Southern Region and East Malaysia. The targeted population is final year social science students. The total size of the target population is 965 students. Data were obtained from stratified random sampling. A total of 333 respondents answered the questionnaire. Based on the questionnaires returned, demographic profile data was tabulated.Section 1 presents information about gender, age, race, marital status and academic qualification. Section 2 assessed the socioeconomic status of the respondents’ parents. Sections 3 and 4 gathered information about respondents’ academic achievement and opinions about their parents’ socioeconomic status affecting their academic achievements. (Table 1)

**Table 1:** Respondents by gender.

Gender	Frequency	Percentage
Male	105	31.5
Female	228	68.5
**Total**	**333**	**100**

From the total respondents (333 people), 68.5% were female, and 31.5% were male. This finding reflects the scenario of student gender in public universities across Malaysia, where the number of female students is twice as high as male students [7]. Table 2 shows that 5 of the respondents (1.5%) were aged 21 years, 92 (27.6%) 22 years, 190 (57.1%) 23 years, 25 (7.5%) 24 years and 1 respondent (0.3%) aged 28 years. Most of the respondents were Malays (78.4%) followed by Chinese (12.3%), India (5.4%) and others 3.9% (Kadazan, Dusun, Iban, Bisaya, dan Bajau; ethnics in Sarawak and Sabah). Meanwhile, the marital status of the respondents was that 98.8% were single, and 1.2% were married. The majority of the respondents (58.6%) have Malaysian Higher School Certificate (STPM) or equivalent qualifications, followed by 101 (30.3%) with matriculation qualifications or equivalent, and 37 (11.1%) with diploma qualifications. (Table 3, Table 4, Table 5, Table 6, Table 7, Table 8, Table 9, Table 10, Table 11)

**Table 2:** Respondents by age.

Age	Frequency	Percent
21	5	1.5
22	92	27.6
23	190	57.1
24	25	7.5
25	20	6.0
28	1	0.3
**Total**	**333**	**100**

**Table 3:** Respondents by race.

Race	Frequency	Percent
Malay	261	78.4
Chinese	41	12.3
Indian	18	5.4
Others	13	3.9
**Total**	**333**	**100.0**

**Table 4:** Respondents by marital status.

Marital status	Frequency	Percent
Single	329	98.8
Married	4	1.2
**Total**	**333**	**100.0**

**Table 5:** Respondents by academic qualification.

Education level	Frequency	Percentage
Matriculation	101	30.3
STPM	195	58.6
Diploma	37	11.1
**Total**	**333**	**100.0**

**Table 6:** Locality of parent.

Locality	Frequency	Percent
Eastern Region	183	55.0
Western Region	60	18.0
Northern Region	43	12.9
Southern Region	38	11.4
East Malaysia	9	2.7
**Total**	**333**	**100.0**

**Table 7:** Employment sector of parent.

Sectors	Father		Mother	
	Frequency	Percent	Frequency	Percent
Private	64	21.5	32	10.0
Government	48	16.1	32	10.0
Self-Employed	131	44.0	44	13.7
Not Working	15	5.0	195	60.7
Others	40	13.4	18	5.6
Total	**298**	**100.0**	**321**	**100.0**

**Table 8:** Income level of parent.

Income level	Father	Mother
B40	88.6	91.8
M40	11.4	7.7
T20	0.0	0.5
**Total**	**100.0**	**100.0**

**Table 9:** Parent's socioeconomic status affecting student achievement.

Answer	Frequency	Percentage
Yes	122	36.6
No	211	63.4
**Total**	**333**	**100.0**

**Table 10:** Parent's socioeconomic status affecting student achievement (Yes).

Factors	Frequency	Percentage
Motivation	71	62.3
Commitment	43	37.7
**Total**	**114**	**100.0**

**Table 11:** Parent's socioeconomic status affecting student achievement (No).

Factors	Frequency	Percentage
Behaviour	142	78.5
Other factors	39	21.5
**Total**	**181**	**100.0**

**Fig. 1:**
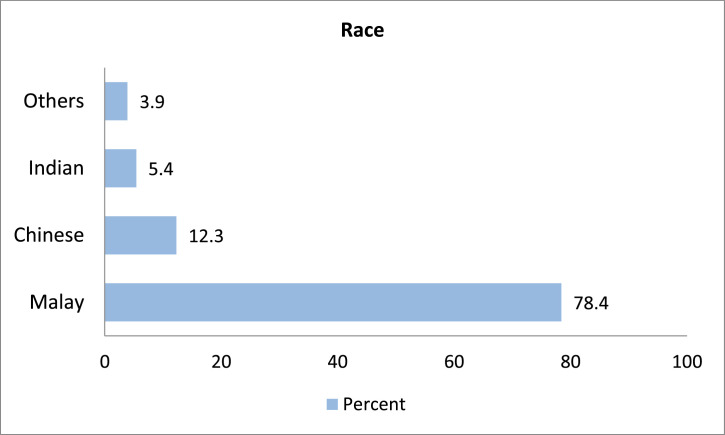
Respondents by ethnic groups.

**Fig. 2:**
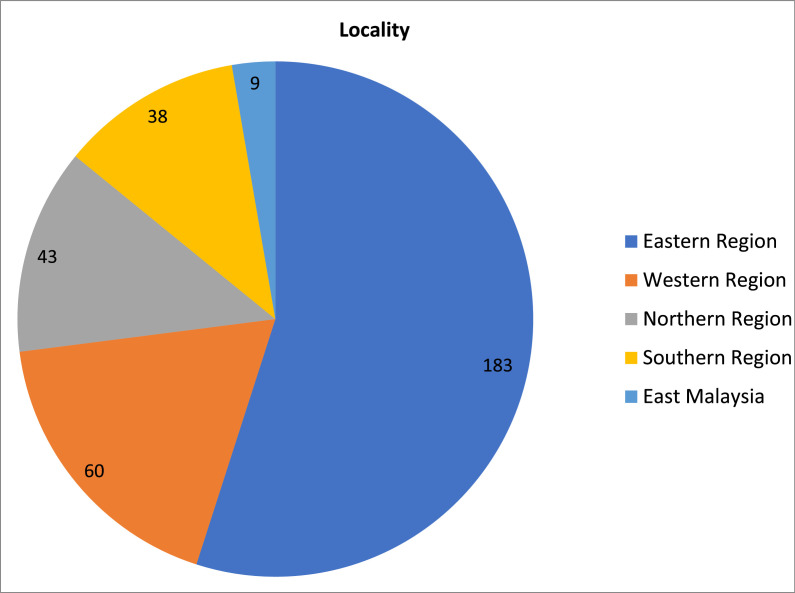
Parent's locality.

**Fig. 3:**
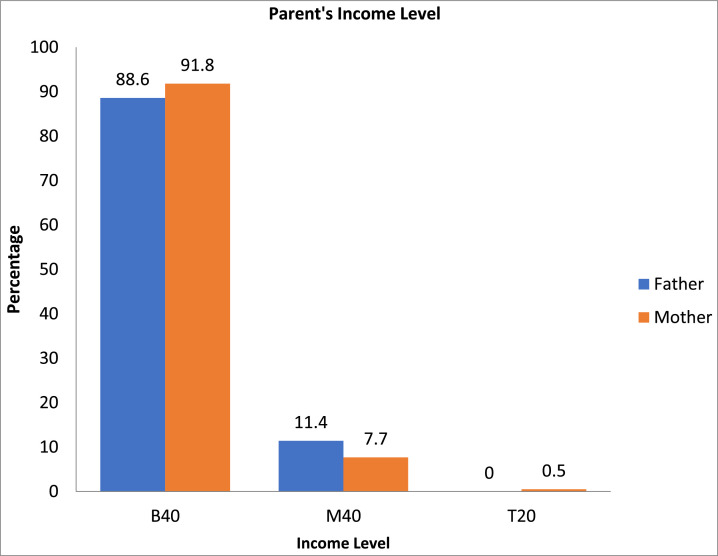
Parent's income level.

The highest locality of respondents’ parents is from the Eastern region (Pahang, Kelantan and Terengganu) by 55%. The locality of parents from the Western region (Perak, Kuala Lumpur, Selangor and Putrajaya) is 18%. The Northern region (Kedah, Penang and Perlis) is 12.9% and the Southern region (Johor, Malacca and Negeri Sembilan) 11.4% while those from East Malaysia is 2.7%. (Figs. 1–3)

The father's employment sector showed that most of the respondents were self-employed with 44.0% followed by working in the private and government sectors with 21.5% and 16.1% respectively. The other sectors (retirees) accounted for 13.4% while the unemployed was only 5.0%. The mother's employment sector showed that most mothers were unemployed (60.7%) followed by self-employed mothers 13.7%. Mothers who work in the private and government sectors recorded a similar percentage of 10.0% while other sectors (retirees) were only 5.6%.

Household income is divided into three categories: B40, M40 and T20 [8]. B40 refers to incomes below RM4,360 (USD1,008). Subsequently, the M40 category earns between RM4,361 (USD1,009) and RM9,619 (USD2,224). While the T20 category income level is above RM9,620 (USD2,225). Father's income for the B40 category was 88.6% followed by M40 which was 11.4%. There is no T20 category for father's income. The mother's income in the B40 category was 91.8% followed by M40 (7.7%). Maternal income for the T20 category was 0.5%.

In total, 211 students or more than 50% of students conclude that parents’ SES does not influence them in their academic achievement nor factor in their future success. Meanwhile, 36.6% of respondents said that parents’ SES influenced their academic achievement. For students who said 'yes', they are motivated by parents' SES to succeed academically. In addition, parents' SES served as a trigger for their commitment to academic achievement. For the students who said 'no', they stated that parents' SES influenced their behaviour and other factors such as mindset, passion and self-motivation to succeed in life.

## 2 Experimental design, materials, and methods

The researcher adopted a survey research design to obtain data from 333 respondents from 14 states in Malaysia, categorised into five regions. Data were gathered utilising a semi-structured questionnaire (Appendix 1). The questionnaire was divided into several sections. Section 1 was used to obtain demographic information from respondents. Section 2 assessed the socioeconomic status of the respondents’ parent. Sections 3 and 4 gathered information about respondents’ academic achievement and opinions about their parents’ socioeconomic status affecting their academic achievements. Ethical consideration in the research process was ensured because administering the questionnaires to respondents was based on their willingness to respond to the research instrument.

## Declaration of Competing Interest

The authors declare that they have no known competing for financial interests or personal relationships that could have appeared to influence the work reported in this paper.

## Appendix Supplementary materials

Image, application 1

Image, application 2
