# Supplementary material for: Data on the impact of socioeconomic status on academic achievement among students in Malaysian public universities
Source: Data Brief. 2020 Jul 14;31:106018. doi: 10.1016/j.dib.2020.106018 (PMC7381507; doi:10.1016/j.dib.2020.106018)
Supplement: Supplementary file 2 [file mmc2.docx]

|  |  |  |
| --- | --- | --- |

**Respondent Code:**


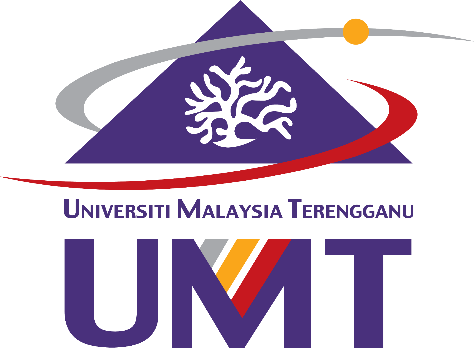


**IMPACT OF PARENTS' SOCIOECONOMIC STATUS ON STUDENT'S ACADEMIC ACHIEVEMENTS**

Dear Sir / Madam,

It is a great pleasure for you to give the cooperation and spend the time to fill in information required in this questionnaire. The purpose of this study is to obtain information on 'IMPACT OF PARENTS' SOCIOECONOMIC STATUS ON STUDENT'S ACADEMIC ACHIEVEMENTS. All information obtained is confidential and may not be disclosed to any third party. Your cooperation is very much appreciated.

Research objective:

- To study the socioeconomic profile of university students in Malaysia.
- To analyze the impact of socioeconomic status of parents on the academic achievement of universities’ students.

| Date of interview : _______________________  Numerator Name : _____________________________________________________ |
| --- |

# SECTION A: RESPONDENT INFORMATION

1. Sex

|  | Male |  | Female |
| --- | --- | --- | --- |

1. Age:
2. Race

|  | Malay |
| --- | --- |
|  | Chinese |
|  | Indian |
|  | Others (Please state: _____________________ |

1. Status

|  | Single |
| --- | --- |
|  | Married |
|  | Others (Please state: _____________________ |

1. Address

                    

State:                                                   

1. Education/Field of study:

1. Academic qualification before entering university

|  | Basic |  |
| --- | --- | --- |
|  | STPM |  |
|  | Diploma |  |
|  | Others (Please state: _____________________ | |

1. Student accommodation during study:

|  | Hostel |  |
| --- | --- | --- |
|  | House/ Room rentals |  |
|  | Family |  |
|  | Others (Please state: _____________________ | |

1. Financial aid for study:

|  | None |  |
| --- | --- | --- |
|  | Scholarship (JPA, etc.) |  |
|  | Loans (PTPTN/MARA) |  |
|  | Parents |  |
|  | Other family member (siblings, etc.) |  |
|  | Others (Please state: _____________________ | |

1. Student spending (scale **1 to 9).** Please select **the priority 1 to 9**.

|  | Payment for rent house/hostel/college | |
| --- | --- | --- |
|  | Transportation (example; petrol/bus/taxi/ LRT) | |
|  | Reference materials/equipment for learning | |
|  | Hand phone post-paid/prepaid | |
|  | Food and drinks | |
|  | Clothing | |
|  | College assignments (example; photostat/binding, etc.) | |
|  | Tuition fees | |
|  | Others (Please state: _____________________ |  |

1. Number of Siblings? _______
2. Rank among siblings? ______

# SECTION B: SOCIO-ECONOMIC STATUS (SES) PARENTS

| **Father Information**   1. Age: 2. Birth place:      1. Job Sector  \|  \| Private \| \| --- \| --- \| \|  \| Government \| \|  \| Self Employed \| \|  \| Not Working \| \|  \| Others (Please state):  _____________________ \| \|  1. Type of job (example: teacher) 2. Main Job: _________________ 3. Side Job: _____________ 4. Monthly Income (RM): 5. Main income: _______________ 6. Side income: _____________ 7. The Highest Education Level:  \|  \| No Education \| \| --- \| --- \| \|  \| UPSR/Standard 6 and Below \| \|  \| PMR/SRP \| \|  \| MCE/SPM/ Secondary School School \| \|  \| Certificate/STPM/STAM \| \|  \| Diploma* \| \|  \| Degree* \| \|  \| Master’s Degree* \| \|  \| Doctor of Philosophy (PhD)* \|  1. (*) Please specify the name of the institution of study and the programs of study 2. Institution:   ________________  ________________   1. Programs of Study:   ________________ | **Mother Information**   1. Age: 2. Birth place:      1. Job Sector  \|  \| Private \| \| --- \| --- \| \|  \| Government \| \|  \| Self Employed \| \|  \| Not Working \| \|  \| Others (Please state):  _____________________ \| \|  1. Type of job (example: teacher) 2. Main Job: _________________ 3. Side Job: _____________ 4. Monthly Income (RM): 5. Main income: _______________ 6. Side income: _____________ 7. The Highest Education Level:  \|  \| No Education \| \| --- \| --- \| \|  \| UPSR/Standard 6 and Below \| \|  \| PMR/SRP \| \|  \| MCE/SPM/Secondary School \| \|  \| Certificate/STPM/STAM \| \|  \| Diploma* \| \|  \| Degree* \| \|  \| Master’s Degree* \| \|  \| Doctor of Philosophy (PhD)* \|  1. (*) Please specify the name of the institution of study and the programs of study 2. Institution:   ________________  ________________   1. Programs of Study:   ________________ |
| --- | --- | --- | --- | --- | --- | --- | --- | --- | --- | --- | --- | --- | --- | --- | --- | --- | --- | --- | --- | --- | --- | --- | --- | --- | --- | --- | --- | --- | --- | --- | --- | --- | --- | --- | --- | --- | --- | --- | --- | --- | --- | --- | --- | --- | --- | --- | --- | --- | --- | --- | --- | --- | --- | --- | --- | --- | --- | --- | --- |

15. Total Parents’ Income (Monthly): RM

**SECTION C: ACADEMIC ACHIEVEMENTS INFORMATION**

1. Cumulative grade point average (CGPA ): _________

2. Grade point average (GPA):_____________

3. Academic achievement awards received (If any)

i. ______________________________________________________________

ii. __________________________________________________________ ___

iii. _____________________________________________________________

iv. _____________________________________________________________

4. What kind of learning mode do you like?

|  | Online Learning |
| --- | --- |
|  | On-campus learning |
|  | Online Learning and Campus Learning |
|  | Others (please state: _____________________ |

1. Do you have a personal computer?

|  | Yes |  | No |
| --- | --- | --- | --- |

1. Are you having problems using your computer?

|  | Yes |  | No |
| --- | --- | --- | --- |

1. Are you having trouble getting information online?

|  | Yes |  | No |
| --- | --- | --- | --- |

1. Do you like buying reference books for courses you take?

|  | Yes |  | No |
| --- | --- | --- | --- |

1. Do you regularly visit the library?

|  | Yes |  | No |
| --- | --- | --- | --- |

1. Do you regularly visit book exhibitions?

|  | Yes |  | No |
| --- | --- | --- | --- |

1. How many hours do you spend on average in reading / revision in a day?

|  | Less than 1 hour |
| --- | --- |
|  | 2-3 hours |
|  | 4-5 hours |
|  | More than 6 hours |

SECTION D: RESPONDENT'S OPINION ABOUT IMPACT OF PARENT'S SSE ON STUDENT'S ACADEMIC ACHIEVEMENTS

**i) PARENT’S EDUCATION LEVEL**

| **No** | **Item** | **Strongly Disagree** | **Disagree** | **Not Sure** | **Agree** | **Strongly Agree** |
| --- | --- | --- | --- | --- | --- | --- |
| 1 | Parent's education level influenced my academic achievement | 1 | 2 | 3 | 4 | 5 |
| 2 | Parent's education level influenced my confidence in academic learning | 1 | 2 | 3 | 4 | 5 |
| 3 | Parent's education level influences my ability to understand a course / subject | 1 | 2 | 3 | 4 | 5 |
| 4 | Parent's education levels influence my efforts to improve academic excellence | 1 | 2 | 3 | 4 | 5 |
| 5 | Parent's education level affects my thinking ability in academic learning | 1 | 2 | 3 | 4 | 5 |
| 6 | Parent's education level influences my choice of field of study | 1 | 2 | 3 | 4 | 5 |

**ii) PARENT’S EMPLOYMENT**

| **No** | **Item** | **Strongly Disagree** | **Disagree** | **Not Sure** | **Agree** | **Strongly Agree** |
| --- | --- | --- | --- | --- | --- | --- |
| 1 | Parent's employment influenced my academic achievement | 1 | 2 | 3 | 4 | 5 |
| 2 | Parent's employment influenced my confidence in academic learning | 1 | 2 | 3 | 4 | 5 |
| 3 | Parent's employment influences my ability to understand a course / subject | 1 | 2 | 3 | 4 | 5 |
| 4 | Parent's employment influence my efforts to improve academic excellence | 1 | 2 | 3 | 4 | 5 |
| 5 | Parent's employment affects my thinking ability in academic learning | 1 | 2 | 3 | 4 | 5 |
| 6 | Parent's employment influences my choice of field of study | 1 | 2 | 3 | 4 | 5 |

**iii) PARENT’S MONTHLY INCOME**

| **No** | **Item** | **Strongly Disagree** | **Disagree** | **Not Sure** | **Agree** | **Strongly Agree** |
| --- | --- | --- | --- | --- | --- | --- |
| 1 | Parent's monthly income influenced my academic achievement | 1 | 2 | 3 | 4 | 5 |
| 2 | Parent's monthly income influenced my confidence in academic learning | 1 | 2 | 3 | 4 | 5 |
| 3 | Parent's monthly income influences my ability to understand a course / subject | 1 | 2 | 3 | 4 | 5 |
| 4 | Parent's monthly income influence my efforts to improve academic excellence | 1 | 2 | 3 | 4 | 5 |
| 5 | Parent's monthly income affects my thinking ability in academic learning | 1 | 2 | 3 | 4 | 5 |
| 6 | Parent's monthly income influences my choice of field of study | 1 | 2 | 3 | 4 | 5 |

**iv** ) Do you agree that your parents' socioeconomic status affects your academic performance at university?

|  | Yes |
| --- | --- |
|  | No |

Explain why?

_______________________________ _________________________________

____________________________________________ ____________________
